# New methods for next generation sequencing based microRNA expression profiling

**DOI:** 10.1186/1471-2164-11-716

**Published:** 2010-12-20

**Authors:** Henk PJ Buermans, Yavuz Ariyurek, Gertjan van Ommen, Johan T den Dunnen, Peter AC 't Hoen

**Affiliations:** 1 Center for Human and Clinical Genetics, Leiden University Medical Center, Einthovenweg 20, 2333 ZC Leiden, The Netherlands; 2 Leiden Genome Technology Center, Leiden University Medical Center, Einthovenweg 20, 2333 ZC Leiden, The Netherlands

## Abstract

**Background:**

MicroRNAs are small non-coding RNA transcripts that regulate post-transcriptional gene expression. The millions of short sequence reads generated by next generation sequencing technologies make this technique explicitly suitable for profiling of known and novel microRNAs. A modification to the small-RNA expression kit (SREK, Ambion) library preparation method for the SOLiD sequencing platform is described to generate microRNA sequencing libraries that are compatible with the Illumina Genome Analyzer.

**Results:**

High quality sequencing libraries can successfully be prepared from as little as 100 ng small RNA enriched RNA. An easy to use perl-based analysis pipeline called E-miR was developed to handle the sequencing data in several automated steps including data format conversion, 3' adapter removal, genome alignment and annotation to non-coding RNA transcripts. The sample preparation and E-miR pipeline were used to identify 37 cardiac enriched microRNAs in stage 16 chicken embryos. Isomir expression profiles between the heart and embryo were highly correlated for all miRNAs suggesting that tissue or cell specific miRNA modifications do not occur.

**Conclusions:**

In conclusion, our alternative sample preparation method can successfully be applied to generate high quality miRNA sequencing libraries for the Illumina genome analyzer.

## Background

MicroRNAs (miRNA) are non-coding RNA transcripts with average length of 21 nt. They play important roles during post-transcriptional regulation of gene expression in various organisms and tissues. Since their discovery by Lee et al [1] the cellular miRNA repertoire has expanded rapidly culminating in over 10,000 entries in the September 2009 miRBase database release [2]. Primary miRNA transcripts are predominantly RNA polymerase II derived strands that contain stem loop hairpin structures. These hairpins get excised from the transcripts by Drosha/DGCR8 in the Microprocessor complex and are transported into the cytoplasm as miRNA precursor miRNA (pre-miRNA) transcripts. The RNase III endoribonuclease Dicer subsequently cleaves the pre-miRNA to release short double-stranded miRNA fragments of on average 21 nt in length. It is generally thought that one of the strands is bound to an Ago protein and incorporated into the RNA induced Silencing Complex (RISC) to act as a guide strand, while the other strand is degraded. Selection of the strands is thought to depend on the relative thermodynamic stability of the ends of the duplex. However, for some miRNAs both strands are detected at comparable expression levels. Once incorporated into a RISC complex, the miRNA strand guides the complex by imperfect base pairing to its targets (reviewed by Kim et al [3]).

Despite their small size, low abundant expression and lack of unifying structural features that allow for selective isolation and/or manipulation, different methods have been developed to measure their expression [4]. A recent addition to the existing miRNA expression profiling techniques is high throughput sequencing. The millions of short sequence reads generated by Next Generation Sequencing (NGS), like the SOLiD (AppliedBiosystems) and Illumina Genome Analyzer, are particularly useful for small RNA transcription profiling. NGS provides miRNA expression profiling at an unprecedented sensitivity and resolution. Compared to available miRNA microarray platforms, the NGS systems are not limited by a predefined number of features, probe design, probe cross hybridization or array background issues. Moreover, the NGS systems directly count the number of transcripts found as a measure for expression abundance, have high multiplexing potential, are species independent, show high sensitivity towards low abundant transcripts and display excellent reproducibility [5].

Different microRNA sequencing library preparation methods have been described. The Illumina genome analyzer protocols for miRNA expression profiling are based on the Modban [6] method. In a comparison by Linsen et al [7], the Small RNA Expression Kit (SREK, Appliedbiosystems/Ambion) showed better between-sample reproducibility compared to both the Modban adaptor (IDT) ligation [6] and poly(A) tailing [8] methods. Although all three methods showed specific systemic biases, these were highly reproducible and therefore do not impair relative miRNA quantification. In the present paper, we present a modified version of the SREK, originally designed for the SOLiD system, for generating high quality miRNA sequencing libraries that are compatible with the Illumina Genome Analyzer technology. The SOLiD Total RNA-Seq kit which has replaced the SREK kit, is based upon the same principles for library generation. The modifications described here can therefore be applied to the new kit as well in order to generate smallRNA, and potentially RNA-seq, libraries that are compatible with the Illumina sequencing platform.

NGS for miRNA expression profiling is becoming a more widely used technology in various biological settings, e.g., hESC differentiation into embryoid bodies [9], leukemia progression [10], chicken [11-13], pig tissues [14], cardiac hypertrophy [15], but also in fundamental miRNA biogenesis studies [16]. Although general guidelines on data processing procedures have recently been proposed [17], downstream data analysis tools for miRNA data, as for all NGS applications, are still in their infancy. Few applications [18-20] that cover all steps involved in miRNA sequencing data analysis, like adapter sequence removal, genome alignment and transcript annotation, in one single analysis pipeline have been described to date. In order to efficiently exploit the massive amounts of data generated by miRNA sequencing platforms, user friendly and easy to use tools need to be developed that report data in intuitive and comprehensive format. In this paper we describe E-miR, a perl-based miRNA sequence data analysis pipeline that combines all individual data handling steps. We apply both the E-miR pipeline and the sample preparation protocol to define cardiac enriched miRNA expression in chicken embryonic development.

## Results

### small RNA library preparation

**Figure 1:**
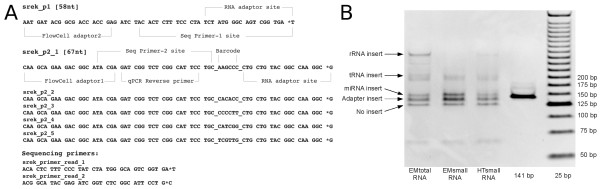
**Amplification primers**. A: Alternative primers used during miRNA sequencing library preparation to make the SREK protocol compatible with the Illumina Genome Analyzer. * Indicates a phosphorothioate bond. B: Library fragment separation on a 6% PAGE gel. Different library fragment sizes can be discerned which represent different types of RNA ligated in between the SREK adapters as indicated at the left of the gel. Due to the length of the alternative primers used during library amplification, the size of an empty library fragment is 125 bp. The miRNA fraction of the library is located at 140-150 bp.

In order to determine cardiac enriched miRNA and isomir expression profiles in stage HH16 chicken embryos, RNA was isolated from the chicken embryonic tissues and integrity was confirmed using Agilent Bioanalyzer pico-RNA and smallRNA-chips. MiRNA enriched RNA fractions (120 ng) from stage HH16 whole embryos (EMs) and Heart tubes (HTs) or 500 ng total RNA from whole embryos (EMt) were ethanol precipitated and dissolved in 3 *μ*L nuclease free water and subsequently used to prepare the miRNA sequencing libraries using the SREK method (Appliedbiosystems/Ambion) with modifications to the manufacturers protocol as described in the Methods section. The libraries were pre-amplified using primers containing sequences that make the SREK libraries compatible with the Illumina flow cell (Figure 1A). The 140-150 bp band that contains the microRNA fraction of the library was excised from a 6% PAGE separation gel, purified and quantified. A representative gel image is shown Figure 1B. MicroRNAs 20b, 206 and 133 have known expression patterns between the tissues [21]. qPCR analysis performed directly on the sequencing libraries for these transcripts confirmed that the miRNA libraries were representative for the biological input samples (Additional file 1 Figure S1). During the preparation of this manuscript, the SREK kit was discontinued and replaced by the SOLiD Total RNA-Seq kit. The adapter sequences used in both these kits are compatible with the PCR amplification primer described here, thus the replacement kit can also be used to generate smallRNA libraries that are compatible with the Illumina platform which it was not initially designed to do.

### Sequence data processing & transcript annotation using the E-miR pipeline

**Figure 2:**
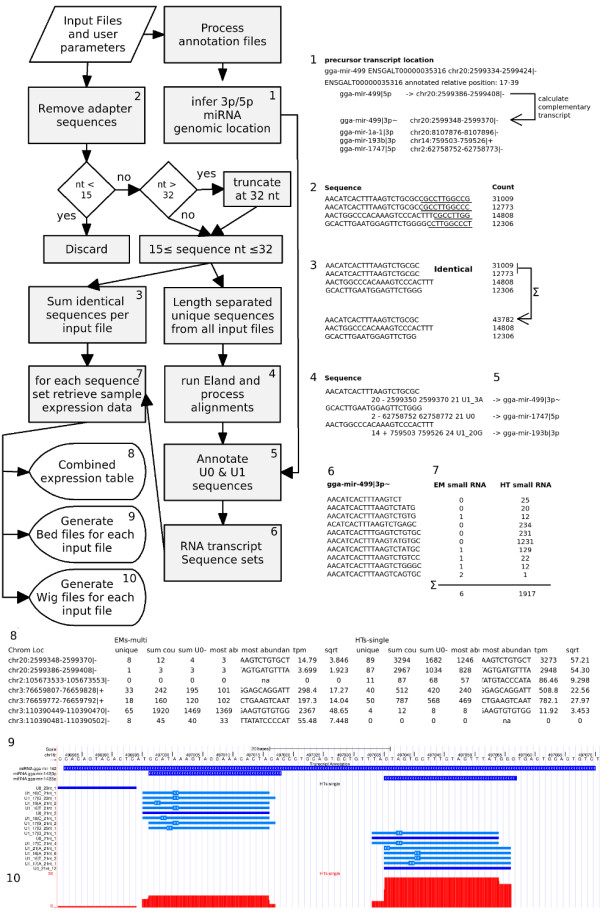
**E-miR pipeline Flowchart**. Schematic overview of the E-miR pipeline. A more detailed description for each of the individual steps can be found in the Methods section.

To facilitate processing of the vast amount of data produced during next generation microRNA sequencing we designed an easy to use perl analysis pipeline called E-miR. A schematic flowchart describing the pipeline is shown in Figure 2. Although it is command line based, the user input is simplified to preparing a file containing a set of run parameters for adapter removal, sequence aligner executable, genome index location and RNA transcript annotation files. Multiple data files can be entered at once. Running the pipeline takes just one command.

Each of the libraries yielded approximately 6 million quality filtered sequences. For removing of 3' adapter sequences, the first 8 nucleotides, CGCCTTGG, were used while allowing for 1 nucleotide mismatch. After adapter removal 2,814,292, 2,926,686 and 3,490,514 sequences with lengths of 15 up to 32 nucleotides remained for the EMt, EMs and HTs libraries, respectively. Overall, 74 to 81% of these sequences could be mapped to the unmasked Gallus gallus-2.1, May 2006 chicken genome release, 29 to 40% of which uniquely aligned with a maximum of 1 mismatch, 34 to 40% were mapped to repeat regions and 5% were uniquely aligned with 2 mismatches. The complete overview of sequence processing and alignment is given in Table 1. The sequences that could not be aligned to the genome may be derived from the unsequenced chicken chromosomes or may be aligned to the reference genome with more than 2 mismatches due to post-transcriptional RNA editing and/or nucleotide additions at the 3' or 5' end of the transcripts.

**Table 1:** EmiR data processing table

	EMtot-single	EMs-single	EMs-multi	HTs-single	HTs-multi
Input	5,794,222	5,294,849	4,042,101	6,517,852	3,292,684
< 15 nt	2,979,930	2,368,163	2,011,435	3,027,338	1,596,093
≥15 nt	2,814,292	2,926,686	2,030,666	3,490,514	1,696,591

NM	577,710	569,163	294,637	921,828	467,263
R*	967,090	1,138,353	814,299	1,383,114	653,343
U0	684,178	571,752	477,298	578,162	295,420
U1	439,677	472,181	333,808	428,136	201,149
U2	145,637	175,237	110,624	179,274	79,416

U0&U1	1,123,855	1,043,933	811,106	1,006,298	496,569

miRNA	557,167 [49.58%]	765,899 [73.37%]	586,779 [72.34%]	442,242 [43.95%]	215,293 [43.36%]
miscRNA	2,934 [0.26%]	1,341 [0.13%]	981 [0.12%]	3,590 [0.36%]	1,849 [0.37%]
pseudogene	10 [0%]	14 [0%]	6 [0%]	17 [0%]	8 [0%]
rRNA	260 [0.02%]	2,392 [0.23%]	1,990 [0.25%]	1,738 [0.17%]	869 [0.18%]
snRNA	366 [0.03%]	360 [0.03%]	285 [0.04%]	614 [0.06%]	344 [0.07%]
snoRNA	45,052 [4.01%]	22,576 [2.16%]	14,706 [1.81%]	32,513 [3.23%]	15,284 [3.08%]
tRNA	10,031 [0.89%]	13,908 [1.33%]	11,625 [1.43%]	27,266 [2.71%]	13,998 [2.82%]
other	508,035 [45.2%]	237,443 [22.75%]	194,734 [24.01%]	498,318 [49.52%]	248,924 [50.13%]

Overview of sequence data processing by the perl analysis pipeline for the heart tube and embryo samples for the single read (single) and multiplexed (multi) libraries. Total RNA input and smallRNA input samples are indicated with the 'tot' and 's' suffixes, respectively. Indicated from top to bottom are the total number of input sequence reads, sequence reads that were shorter than 15 nt after 3' adapter truncation, sequences accepted for further analysis after 3' adapter truncation, genome alignment classes and the number of sequences annotated to non-coding RNA transcripts. For the genome alignment classes, NM indicates reads that could not be mapped to the genome, R* reads that were mapped to repeat areas and U0-U2, reads that were mapped with either no, one or two mismatches to the genome. Percentages of annotated transcripts are calculated relative to the sum of U0 and U1 reads. The 'other' category indicates reads not annotated to any of the Chicken non-coding RNAs in the Ensembl database.

Only sequence tags uniquely aligned to the genome with a maximum of one nucleotide mismatch were annotated to known non-coding RNA transcripts as annotated in Ensembl. A sequence tag was annotated to a non-coding RNA transcript when there is at least a 50% overlap on genomic positions. In agreement with other miRNA sequencing studies the majority of annotated sequences represented miRNA transcripts, with relatively small contributions from other non-coding RNA species like snoRNA, and tRNAs [9,10]. A summary listing the annotated RNA species distribution is given in Table 1. Not all sequences mapped to known Chicken non-coding transcripts. Approximately 90% of these had expression levels below 5 tpm.

For the remainder of this manuscript, we did not use the mature/star nomenclature as these suggests that the miRNA transcript annotated as the mature form is bound to the Ago protein within the RISC complex and has more abundant expression than the star, while this is not always true. Moreover, the miRbase and Ensembl databases may not list information on both the mature and star transcripts from one precursor, impairing mature/star annotation. Instead, we chose to name the miRNA transcript according to the 5-prime (5p) or 3-prime (3p) arm of the hairpin they originated from. This nomenclature is already used in cases where two different miRNA dicer products were detected from opposite arms of the the same precursor transcript [22]. In cases where annotation for only one of the 5p or 3p Dicer cleavage products was available from the Ensembl database, the complementary sequence positions were inferred as described in the Methods section. Sequencing data has shown that miRNA precursor may give rise to multiple miRNA Dicer products which vary in length and nucleotide compositions [9,10]. These length variations impair estimation of genomic positions of the 5p and 3p miRNA transcript pairs that are derived from the precursor hairpin. In order to cope with this, EmiR allows for a minimal 50% overlap on genomics positions of the sequence read and the miRNA loci for annotation of the sequence reads.

**Figure 3:**
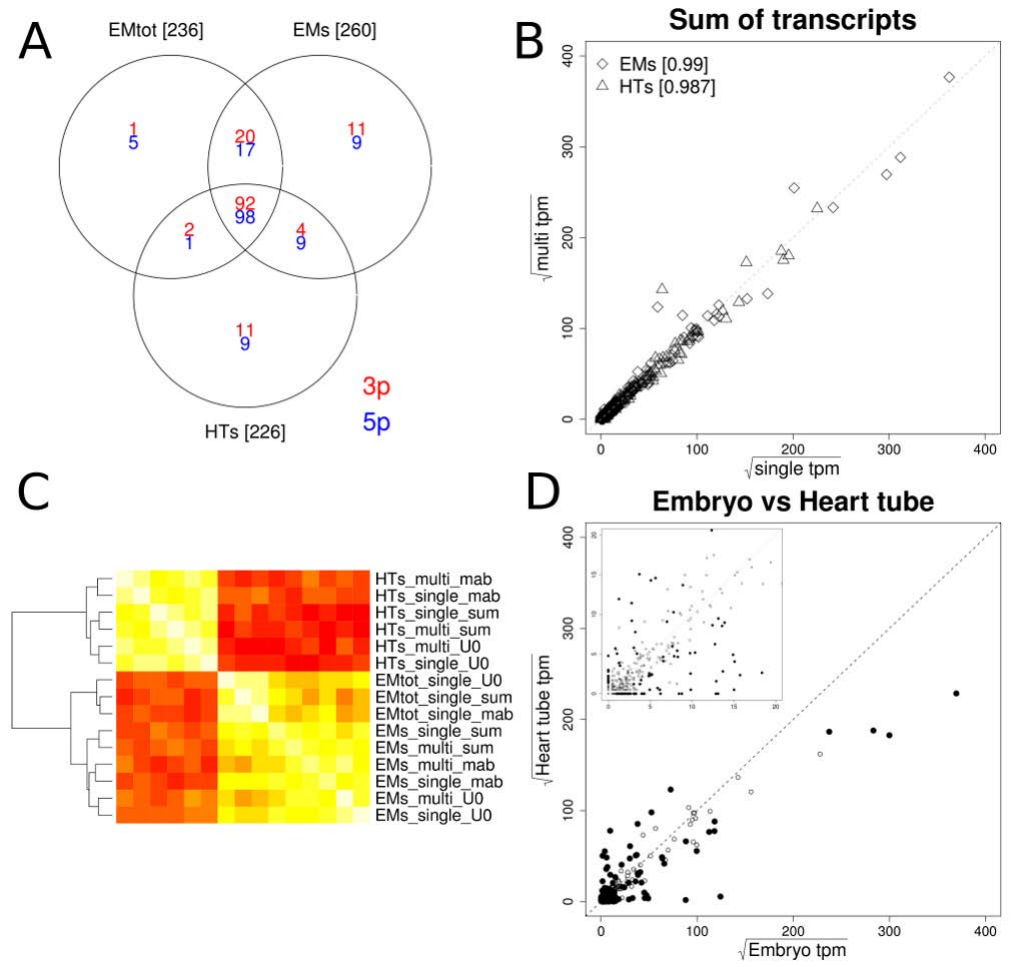
**EM and HT miRNA expression**. A: Venn diagram indicating the number of 5p (blue) and 3p (red) miRNA transcripts expressed with at least 5 tpm in either HT or whole embryo libraries. B: Scatter plots comparing the single (x-axis) and multiplex (y-axis) sequencing run for the sum of isomirs per transcripts to calculate miRNA transcript expression levels in sqrt(tpm). Heart tube and Embryo samples are indicated with triangles and diamonds, respectively. Pearson correlation coefficients are indicated at the top of the plot for each summation method. C: Heatmap clustering comparing all samples used in this study. All expressed miRNA transcripts were used to generate this heatmap. Indicated at the right are the tissues (EM & HT), total (tot) or small enriched (s) RNA, the single or multiplex runs. The different summation methods to calculate miRNA transcript expression levels for each sample, i.e., the sum of all isomirs, the sum of uniquely aligned isomirs without mismatches and the most abundant isomir, are indicated with '_sum', '_U0' and '_mab', respectively. Horizontal and vertical labels are identical. D: Scatter plot indicating average miRNA expression in sqrt(tpm) for heart tube (x-axis) and whole embryo (y-axis). Open and closed black circles represent non-significant and significantly differentially expressed miRbase miRNA transcripts respectively. The top left insert depicts an enlarged section of the 0-20 sqrt(tpm) area.

Dicer processing of the 560 chicken miRNA precursor transcripts listed in the Ensembl database (v56) lead to 1120 possible 5p and 3p miRNA transcript pairs. In total 289 of all 1120 possible unique 5p and 3p miRNA transcripts were expressed with at least 5 transcripts per million (tpm), of which 236, 260 and 226 miRNA transcripts in the EMt, EMs and HTs libraries, respectively (Figure 3A). The sequence data provided evidence of expression for 88 5p and 93 additional 3p transcripts not listed in the Ensembl database. These transcripts are indicated with the '~' preceded by the 5p/3p annotation in the expression table. The E-miR output data table for all five input samples is available in Additional file 2 Table S1.

One of the features of the SREK kit and its replacement SOLiD Total RNA-Seq kit is that both retain the strandedness of the inserted RNA transcripts in the library. Almost all of the sequencing reads that are annotated to non-coding RNA transcripts are sense, with only 0.017% in the antisense orientation. For a subset of microRNAs, low abundant sequence reads mapping to other Dicer cleavage fragments, like the head of the hairpin structure and the regions immediately flanking the precursors were detected, albeit with low expression levels.

### Highly correlated multiplex and single run expression profiles

The NGS platforms have a high multiplexing potential. In order to investigate multiplexing performance of the modified library preparation method, the individual sequencing libraries were constructed with a unique 6 nucleotide sequence bar-code positioned in the reverse primer during library pre-amplification. A multiplexed pool consisting of equimolar amounts of the smallRNA derived EM and HT libraries was prepared. After sequencing, the sequence reads were separated on sample origin based on perfect matches of the 6 nt multiplex tags. The multiplexed libraries showed highly similar data processing and transcript annotation characteristics compared to the the single runs as is evident from Table 1 and the scatter plot in Figure 3B.

For reasons unknown, gga-mir-92-3p expression levels deviated from expected in all multiplex libraries. Excluding these transcripts further increased Pearson correlations coefficients up to 0.995. For virtually all miRNA transcripts detected, multiple sequence variants, i.e., isomirs, could be observed in the data, reaching up to 1002 unique sequence variants for the 'miRNA|ENSGALT00000042432|5p||sense' transcript in the HT and EM samples. The number of unique isomirs for each miRNA per sample is listed in the E-miR expression output table. Different summation methods to calculate the expression level for each miRNA transcript from the individual isomir complement have been proposed. In agreement with previous reports, the sum of individual reads, the most abundant isomir read and the sum of isomirs aligned uniquely without mismatches, were highly correlated [9,10]. Figure 3C shows a heatmap clustering based on Pearson correlation coefficients for the miRNA expression levels calculated from the sum of individual reads, the most abundant read and the sum of isomirs aligned uniquely without mismatches for both the multiplex and single sequencing runs used in this study. A clear separation between the heart tube and embryos is evident with a sub division between the Embryo libraries derived from small RNA enriched and total RNA. The E-miR pipeline reports all three expression values per transcript. For the remainder of this manuscript the sum of all isomirs per miRNA will be referred to as the expression of a specific miRNA transcript. In conclusion, our miRNA sequencing library preparation methods allows for reliable sample multiplexing.

### Choice of RNA input type affects the miRNA expression profile

For the whole embryo, both total RNA and smallRNA enriched RNA fractions, originating from the same sample homogenate, were used to generate small RNA sequencing libraries. Overall sequence alignment characteristics were similar between the two RNA types. However, they did show a striking difference in the total number of annotated sequences, i.e., approximately 45 and 23% for the smallRNA enriched fraction and totalRNA based libraries, respectively (Table 1). MicroRNA transcripts were the main component of this difference, representing approximately 50 and 73% of sequences for the totalRNA and smallRNA libraries, respectively. Also, in the small RNA derived sample, more miRNA transcripts were expressed above 5 tpm (Figure 3A) and in a Limma analysis comparing the small and total RNA derived Embryo samples, 6 miRNAs were shown to have significantly higher expression levels for the small RNA derived libraries (Additional file 3 Figure S2). These data indicate the choice of RNA input affects the miRNA expression profile.

### Shorter pre-amplification primers increase miRNA sequencing depth

**Figure 4:**
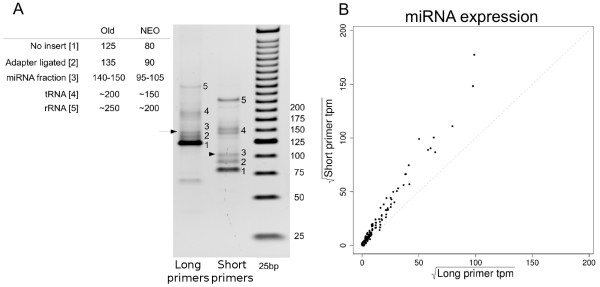
**Short amplification primer libraries**. A: 4-12% PAGE gel visualizing improved library fraction separation by using the shorter amplification primers. B: Scatter plot highly correlated miRNA expression levels [sqrt(tpm)] between libraries generated using the long (x-axis) and short (y-axis) amplification primers. Expression levels were calculated relative to the sum of aligned transcripts.

A crucial step to produce high quality microRNA sequencing (miSeq) libraries is the PAGE size selection at which the miRNA containing library is separated from library fractions containing interfering library inserts, like the adapters that were ligated in between adapters and adapters ligated without RNA inserts. The physical separation of these fractions on the PAGE gel is evident, but with a small margin for error in when excising the 140-150 bp section from the gel. This is what caused the relatively large fraction number of reads that were discarded during adapter trimming at the 3' end of the reads. A set of shorter alternative amplification primers was designed (Additional file 4 Figure S3) that yield an overall shorter library size to 95-105 bp, which improves the fraction separation, reducing unwanted fractions from the microRNA library and increasing miRNA sequencing depth (Figure 4A). Expression profiles of libraries generated with the long or short primer set were highly correlated (Pearson correlation coefficient = 0.989). Moreover, for all miRNA transcripts higher miRNA expression levels were detected when using the short primers, clearly indicating their improved selectivity (Figure 4B).

### Differential miRNA transcript expression

For statistical analysis of differential miRNA expression between the heart and embryo the single and multiplex sequencing runs were treated as technical replicates in the Limma based statistical analysis for differential expression. To normalize the data, the tpm values were square root transformed. In total 152 miRNA transcripts were differentially expressed with BH-FDR p-values ≤ 5% and at least a 1.5 fold difference between the small RNA enriched heart tube and embryo libraries. A scatter plot depicting these results is shown in Figure 3D. After filtering for transcripts with ≥ 5 tpm in either group, 115 remained. Of these 37 showed higher expression in the heart (Table 2). Previously described cardiac enriched microRNAs 499-5p&3p [23] and miR families 1 and 133 [21] constituted the top of the list. In addition to these transcripts our data list microRNAs 490-5p&3p, 1677-5p, 126-5p, 1434-5p, 193b-3p, and the members of the miR30 family, i.e., 30a-5p, 30b-5p, 30d-5p and 30e-5p, as novel cardiac enriched transcripts. In contrast 3p and 5p transcripts for miRNAs 219, 122-1 and 10b and 206 showed depleted expression in the embryonic heart. Additional file 5 Table S2 lists all Limma results.

**Table 2:** Cardiac enriched miRNAs

miRNA transcript	Location	EM-s	HT-s	Fold Difference
miRNA*|*ENSGALT00000028994*|*5p~|gga-mir-133a-1*|*sense #	chr2:105670407-105670428*|*-	0 ± 0	9 ± 1.3	inf
miRNA*|*ENSGALT00000028999*|*3p*|*gga-mir-1a-1*|*sense	chr20:8107876-8107896*|*-	0 ± 0	23 ± 6.9	inf
miRNA*|*ENSGALT00000029007*|*5p~*|*gga-mir-133c*|*sense #	chr23:4664062-4664083*|*+	0 ± 0	8 ± 2.9	inf
miRNA*|*ENSGALT00000035316*|*5p*|*gga-mir-499*|*sense #	chr20:2599386-2599408*|*-	2.8 ± 1.3	2542.5 ± 574	906
miRNA*|*ENSGALT00000035316*|*3p~*|*gga-mir-499*|*sense #	chr20:2599348-2599370*|*-	15.5 ± 1.1	3048.4 ± 318.2	196
miRNA*|*ENSGALT00000029007*|*3p*|*gga-mir-133c*|*sense #	chr23:4664099-4664120*|*+	2.8 ± 1.3	496.9 ± 44.9	177
miRNA*|*ENSGALT00000029006*|*5p~*|*gga-mir-1b*|*sense	chr23:4663916-4663936*|*+	1.7 ± 1.1	142.9 ± 22.7	83
miRNA*|*ENSGALT00000028999*|*5p~*|*gga-mir-1a-1*|*sense	chr20:8107838-8107858*|*+	35.4 ± 20.4	2366.1 ± 777.2	67
miRNA*|*ENSGALT00000029006*|*3p*|*gga-mir-1b*|*sense	chr23:4663953-4663973*|*+	91.1 ± 24.2	6086.5 ± 1328.3	67
miRNA*|*ENSGALT00000042439*|*5p*||*sense	chr4:2151195-2151215*|*+	1.4 ± 2	32.5 ± 0.4	23
miRNA*|*ENSGALT00000042439*|*3p*||*sense #	chr4:2151238-2151260*|*+	30.2 ± 6.1	1277.3 ± 123.2	42
miRNA*|*ENSGALT00000035276*|*3p*|*gga-mir-490*|*sense	chr1:59948716-59948737*|*-	45.3 ± 12.6	1447.5 ± 231.3	32
miRNA*|*ENSGALT00000042468*|*5p*|*gga-mir-1773*|*sense	chr20:8109147-8109169*|*+	1.1 ± 0.2	25.5 ± 3.8	23
miRNA*|*ENSGALT00000042438*|*3p*|*gga-mir-1799*|*sense	chr5:42365992-42366012*|*+	8.6 ± 6.9	130.5 ± 0.5	15
miRNA*|*ENSGALT00000035276*|*5p~*|*gga-mir-490*|*sense	chr1:59948755-59948776*|*-	13.8 ± 2.1	228.3 ± 47.4	17
miRNA*|*ENSGALT00000042432*|*3p~*|*sense	chr1:104486649-104486675*|*-	8.2 ± 4.6	84.6 ± 5.7	10
miRNA*|*ENSGALT00000029028*|*3p*|*gga-mir-133b*|*sense #	chr3:110384948-110384968*|*-	93 ± 21.6	866.5 ± 52.1	9.3
miRNA*|*ENSGALT00000042246*|*3p~*|*gga-mir-1731*|*sense	chr12:10938277-10938299*|*-	1.7 ± 1.1	15 ± 1.6	8.8
miRNA*|*ENSGALT00000042411*|*5p*|*gga-mir-1434*|*sense	chr28:1055205-1055224*|*+	26.5 ± 11.3	204.5 ± 18.6	7.7
miRNA*|*ENSGALT00000035271*|*3p*|*gga-mir-193b*|*sense	chr14:759503-759526*|*+	31.8 ± 0.3	213.2 ± 26	6.7
miRNA*|*ENSGALT00000042289*|*5p*|*gga-mir-1747*|*sense	chr2:62758752-62758773*|*-	2.1 ± 1.2	12.5 ± 0.6	6.1
miRNA*|*ENSGALT00000028974*|*5p*|*gga-mir-146a*|*sense	chr13:7555655-7555676*|*-	10.6 ± 4.2	60.6 ± 8.3	5.7
miRNA*|*ENSGALT00000028997*|*5p*|*gga-mir-30d*|*sense #	chr2:148337300-148337321*|*-	1452 ± 39.6	7275.3 ± 46.5	5.0
miRNA*|*ENSGALT00000042432*|*5p*||*sense	chr1:104486710-104486736*|*-	915.9 ± 102.7	3702.3 ± 152.3	4.0
miRNA*|*ENSGALT00000028990*|*3p~*|*gga-mir-138-1*|*sense	chr2:40745167-40745183*|*-	9.4 ± 2.4	33.5 ± 1	3.6
miRNA*|*ENSGALT00000028998*|*5p*|*gga-mir-30b*|*sense	chr2:148331648-148331669*|*-	461.2 ± 63	1638.4 ± 129.7	3.6
miRNA*|*ENSGALT00000029030*|*3p*|*gga-mir-223*|*sense	chr4:233007-233027*|*+	2759.8 ± 88	9608.1 ± 564.1	3.5
miRNA*|*ENSGALT00000042472*|*5p*|*gga-mir-1677*|*sense #	chr3:76659772-76659792*|*+	219.8 ± 31.9	725.3 ± 80.2	3.3
miRNA*|*ENSGALT00000028951*|*5p*|*gga-mir-125b*|*sense #	chr1:102457663-102457684*|*+	5255.7 ± 209.1	15151.5 ± 1241	2.9
miRNA*|*ENSGALT00000035272*|*3p*|*gga-mir-181a-1*|*sense	chr8:2001620-2001641*|*+	154.6 ± 49.6	424.6 ± 8.1	2.7
miRNA*|*ENSGALT00000029026*|*5p*|*gga-mir-30a*|*sense #	chr3:85102244-85102265*|*+	898.4 ± 20.4	2238.4 ± 52.7	2.5
miRNA*|*ENSGALT00000035333*|*3p*|*gga-mir-144*|*sense	chr19:5824134-5824155*|*-	79.4 ± 4.2	187.5 ± 8.9	2.4
miRNA*|*ENSGALT00000029015*|*5p*|*gga-let-7k*|*sense	chr26:1442955-1442976*|*-	11.8 ± 1	25.5 ± 3.8	2.2
miRNA*|*ENSGALT00000028983*|*5p*|*gga-mir-126*|*sense #	chr17:8431792-8431812*|*-	1310.7 ± 123.6	2561 ± 161.5	2.0
miRNA*|*ENSGALT00000029008*|*5p*|*gga-mir-30e*|*sense	chr23:5248432-5248450*|*+	1382.7 *_ *30.5	2630.2 ± 3.1	1.9
miRNA*|*ENSGALT00000042329*|*3p~*|*gga-mir-1811*|*sense	chr4:58651749-58651768*|*+	60.9 *_ *6	105.5 ± 4	1.7
miRNA*|*ENSGALT00000028959*|*3p~*|*gga-mir-18a*|*sense	chr1:152248637-152248658*|*-	49.1 *_ *0.3	82.5 ± 2.8	1.7

Table of significantly cardiac enriched miRNA transcripts. Indicated are transcript name (EnsemblTranscript | 5p/3p | miRNA name | (anti)sense), genomic location, average expression values ± SD and the fold difference between samples. The ' ~' indicates miRNA transcripts for which the genomic positions were not present in the Ensembl database and thus were inferred from the complementary position of the hairpin structure. # indicates the difference in expression level was confirmed by qPCR.

### Quantitative PCR confirmation of microRNA expression profiles

**Figure 5:**
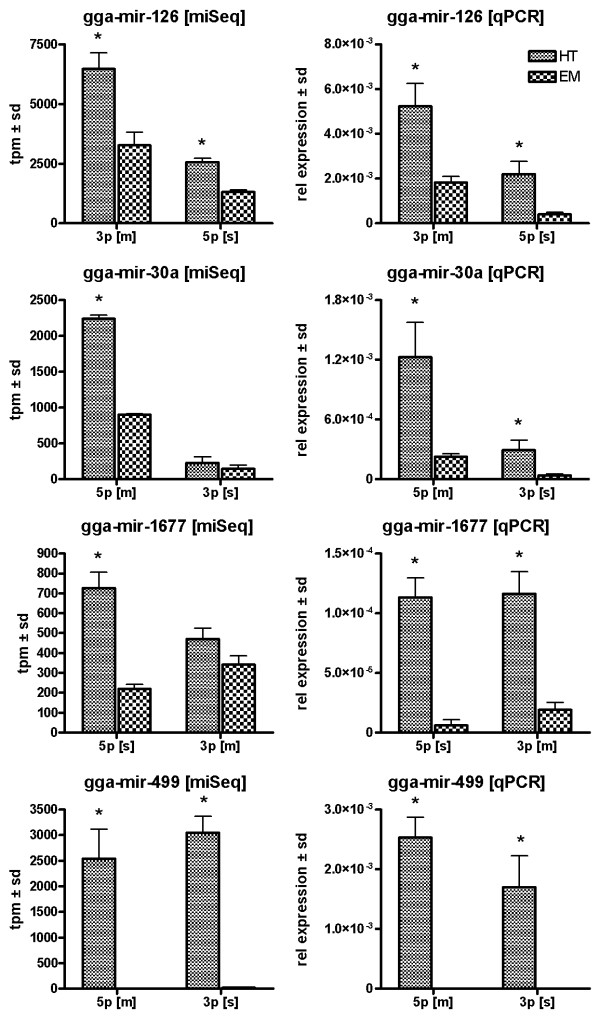
**qPCR confirmation of sequencing data**. MicroRNA expression levels for both 5p and 3p transcripts from single precursor transcripts in HH16 whole chicken embryo and heart tube. 5 s rRNA was used as an internal control to normalize gene expression. Left and right column represent miSeq and qPCR derived expression levels, respectively. The bars represent mean expression levels ± sd. * indicates a significant difference in gene expression relative to whole embryo.

Expression profiles for a subset of cardiac enriched miRNAs was confirmed with qPCR using a set of independent heart tube and embryo samples (Figure 5 and Additional file 6 Figure S4). Our data confirms previously recognized cardiac enriched miRNA expression transcripts like miR499 and the miR133 family. In addition, qPCR confirmed other transcripts showing cardiac enrichment like the miR30a/b/d, miR126 and miRNA ENSGALT0000042439-3p, a transcript which has not yet been included in the miRbase [2]. Lower expression levels in the heart tube were confirmed for miR122-1-5p in addition to miR206-3p. Overall, miSeq and qPCR expression profiles were highly similar. However, a difference in expression for miR1677-3p was not observed between tissues using sequencing, cardiac enrichment was detected using qPCR. In contrast, significant differences in expression for the miR20b-5p transcript could not be confirmed by qPCR.

### miRNAs display uniform differential isomir expression profiles

**Figure 6:**
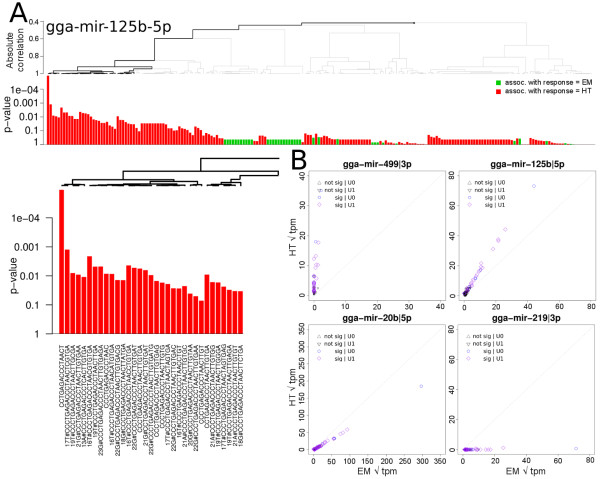
**Globaltest analysis results**. Example covariate plot for the miRNA-125b-5p transcript (A) displaying the individual contributions of its 203 isomirs to the overall test statistics for differential expression. Significantly contributing transcripts are indicated by the dark line in the hierarchical tree. B: Zoomed section of the hierarchical tree depicting the subset of 23 significantly contributing isomir sequences. Positions and the detected nucleotide of variations are indicated, e.g., 17T# indicates that nucleotide 17 in the reference sequence was a 'T'. C: Isomir expression plots between the Heart and Embryo showing uniform differential isomir expression patterns for mir-499-3p, mir-125b-5p, mir-20b-5p and mir-219-3p, respectively.

Multiple sequence variants, i.e., isomirs, were detected for most of the miRNA transcripts. The Limma based analysis for differential expression ignores the heterogeneous isomir complement present for each miRNA transcript and condenses these into one value. We used the Globaltest developed by Goeman et al [24], which tests whether a group of covariates, in this case the isomir complement for each miRNA, is associated with the difference in phenotypes, i.e., HTs vs EMs. Following to the criteria described in the Methods section, 19004 unique isomirs were selected, representing 228 miRNA transcripts. At a BH-FDR cut-o at 5%, 84 miRNAs were significantly differentially expressed between the Heart and the Embryo according to the Globaltest. The Limma analysis had identified 72 of these. To avoid mis interpretation due to stochastic sampling effects of less abundant miRNAs, only miRNAs for which the most abundant isomir was above 50 tpm were taken into consideration for further analyses, leading to 50 miRNAs (Additional file 7 Table S3). Covariate plots that visualize the contribution of the individual isomirs to the test statistics were generated for the significant miRs. An example plot is shown in Figure 6A for miR-125b-5p. Significantly contributing isomirs are indicated by the black line in the hierarchical clustering at the top of the plot. An enlarged section of the significantly contributing isomirs shows the isomir sequence identity. For a subset of microRNAs, a whole branch of the tree of isomirs was considered to contribute significantly as a whole, instead of individual isomirs. This indicates that there is differential expression for that miRNA, but it could not be attributed to any specific set of isomirs. For most of the significant miRNAs the direction of differential expression for the majority of the individual isomirs was identical. Isomirs deviating from this, in general had a low contribution to the test statistic.

To gain more insight into the expression levels of the significantly contributing isomirs, scatter plots were generated that show the individual isomir expression levels for the heart and embryo. Figure 6B shows similar patterns of differential expression for all isomirs related to gga-mir-499-3p, gga-mir-125b-5p, gga-mir-20b-5p and gga-mir-219-3p. These high correlations between isomir expression levels were also observed for the not significantly regulated miRNAs (data not shown). The observation that all isomirs are regulated similarly suggests that there is no tissue specific editing of miRNA transcripts.

In conclusion, the Globaltest can be applied to test for differential transcript expression, while taking the specific isomir complement of the miRNA into account. However, given the highly correlated isomir expression profiles, using either the most abundant transcript or the sum of transcripts as a measure of the miRNA abundance, would essentially lead to similar results with a Limma analysis. The highly correlated isomir expression between the heart and embryo suggest that tissue or cell specific miRNA modifications do not occur.

## Discussion

### miSeq library construction by SREK adaptations

The library preparation methodology we describe is an adaptation on the SREK kit, which was initially designed to prepare miSeq libraries for the SOLiD Next-Generation Sequencing system (Appliedbiosystems), to make it compatible with the Illumina Genome Analyzer technology. Compared to the Modban method on which the standard Illumina library generation protocol is based, the SREK kit was shown to have more preferable reproducibility characteristics [7]. The main modification to the protocol is usage of a set of alternative library amplification primers which contain sequence features compatible with both the adapter sequences that were introduced during the initial adapter ligation as well as the oligonucleotides deposited on the Illumina Genome Analyzer flow cell (Figure 1).

Our libraries were prepared from 500 ng total RNA or 120 ng small enriched RNA fractions. A mere 1 *μ*L of the ligated and reverse transcribed cDNA pool is used during library pre-amplification in a 100 *μ*L reaction volume, which generally yields more than enough library for several sequencing runs. This means that in principle an equivalent of 3 ng of small enriched RNA is sufficient to successfully prepare miSeq libraries with this method. However, no attempts were made to purify and concentrate the cDNA pool in order to significantly reduce the input RNA quantity to these lower ng levels.

Our results point to using the following recommendations for generating high quality miRNA sequencing libraries. 1) Although both small and total RNA can be used, miRNA enriched small RNA fractions libraries showed increased sensitivity towards miRNA expression detection. 2) 100 ng smallRNA enriched RNA. Other miRNA libraries have successfully been generated in our laboratory using as little as 25-50 ng RNA. However, to yield enough library, several parallel pre-amplification reactions may be be needed. 3) The short amplification primers allow for increased selectivity and sensitivity towards miRNA transcripts. Needless to say, identical conditions for all processed samples are recommended.

### Chicken embryonic heart tube enriched miRNA expression

To validate our sample preparation approach, we prepared libraries from chicken HH16 heart tubes and whole embryos without heart tube. Chicken have been used as a model for cardiac developmental biology for many years mainly due to the fact that the heart initially develops outside chest cavity and cardiac development can be precisely timed [25]. Moreover, for some miRNA transcripts cardiac enriched or cardiac restricted patterns during chicken (cardiovascular) embryonic development have previously been described [21], making this an excellent model to validate our approach.

Our statistical analysis of the expression data yielded a total of 37 significantly cardiac enriched miRNA transcripts, containing all of the previously described characteristic cardiac enriched microRNA transcripts, like miR499 and the miR1 and 133 family. Cross referencing our cardiac enriched miRNA transcripts with previously published mouse and human sequencing data generated by Landgraf et al [22] showed overlap for miR144-3p, miR126-5p and 146a-5p. However, overlap between our data and zebra-fish cardiac enriched transcripts was limited to the miR499 and the miR133 family [26], while the Zebra fish cardiac enriched miR221 and miR130b miRNA transcripts showed significantly lower expression in our chicken heart data compared to embryo expression levels. Differences in species and developmental stages are likely to underlie these apparent discrepancies.

A subset of the differentially expressed miRNA transcripts were confirmed with qPCR on a set of biologically independent samples. These included not only the characteristic cardiac miRNAs 133 and 499, but also novel cardiac enriched transcripts miR1677 and ENSGALT0000042439-3p. Differential expression for miR 1799-3p with qPCR could not be confirmed using qPCR since no reliable expression signal could be detected. Given that this transcript had low miSeq expression levels, i.e., approximately 130 tpm, this implies insufficient sensitivity of realtime PCR techniques. Although differences in the absolute expression levels did not perfectly correlate between methodologies, the relative expression patterns did agree as has also been described by Linsen et al. [7]. In conclusion, the chicken cardiac enriched sequencing data generated by our alternative library preparation approach yields biologically valid data.

### miSeq analysis pipeline

Essential to the analysis of miRNA sequencing data is removing any adapter sequences that were introduced during the library preparation as these impair proper genomic alignment. In the E-miR pipeline, the user is required to provide this sequence and state if single nucleotide mismatches are allowed during adapter identification. This stretch of adapter sequence needs to be chosen carefully since matches to the first 4-6 nt of the adapter sequence may occur by chance within the RNA insert and cause aberrant truncation of the sequence. Allowing for mismatches in this step augments this even more, leading to deviating expression profiles. In our experience, using the first 8 nucleotides of the 3' adapter sequence with allowing for one mismatch does not affect the expression profiles. The SeqBuster [20] miRNA analysis pipeline also includes a step to remove 3' adapter sequences from the sequences. For the same dataset, the E-miR pipeline removed the 3' adapter sequences approximately 2-3× faster. More importantly, after inspection of the truncated sequence output, E-miR appeared to be more accurate in identifying and removing of these sequence features. Another advantage of the E-miR over SeqBuster is that multiple data files can be processed simultaneously.

With the increasing amount of data generated by the different NGS systems, there is a need for faster sequence alignment tools. Many, short read aligners have been described, all of which have their specific characteristics [27]. All of the next generation short-read alignment tools available like Eland (part of the standard Illumina analysis pipeline), Rmap [28] and Bowtie [29], outperform Blast [30] on speed. However, these increased alignment speeds affect the quality of the alignments [31]. Unlike Eland, the Rmap and Bowtie aligners are not limited to a predefined 32 nt sequence length. Nevertheless, since microRNAs have an average length of approximately 21 nt, this 32 nt sequence length limitation present in Eland does not pose a bottleneck for microRNA transcript alignment purposes. However, Eland but not Bowtie, is a commercial aligner. To meet the demands for faster alignments and provide more flexibility in alignment settings a Bowtie E-miR version of the pipeline is also available. The Bowtie version did the same job as the Eland version in just 39 min, mainly by reducing the alignment time from 1 hour and 43 min to under 2 min.

### Isomir expression profiles

MiRNA isomirs may be derived by a combination of variable Dicer cleavage points, nucleotide additions and RNA editing. Nucleotide variations to the reference miRNA transcript sequence may have altered target specificity and thereby modulate different biological processes to further fine tune post-transcriptional gene expression. For this to manifest, the isomir should be differential expressed between conditions, and, the expression should not correlate with the overall differential isomir expression pattern that is observed between the groups. Although we identified the specific subset of isomirs that significantly contributed to the differences in expression of each differentially expressed miRNA by exploiting the ability of the Globaltest [24], uniform differential expression was observed for all isomir transcripts between the heart and embryo. This was also seen for the set of not significantly differentially expressed miRNAs. Similar results were obtained when applying the crossmapping correction method as proposed by de Hoon et al [32] in order to handle sequence reads that map to multiple loci. These observations suggest that the mechanisms by which the isomir complement is generated may not be specific to the cell type or differentiation state of the tissue under investigation but rather to represent a general non-tissue dependent system and hence isomir tissue specific post-transcriptional effects not to occur. Further research is needed to ascertain whether the absence of non-uniform isomir expression is a general phenomenon or confined to our sample collection.

To date, only the SeqBuster analysis pipeline describes a method for analyzing isomir complement across and within samples [20]. In the SeqBuster approach, first a subset of isomirs of interest is selected based on common features, e.g., nucleotide modifications. These subsets are subsequently tested for differential expression across the conditions. We chose to take the opposite approach, i.e., the Globaltest first provides insight into which miRNAs are differentially expressed across the groups, based on their isomir complement. Only then are those isomirs identified that significantly contribute to this difference. This is a more unbiased and less hypothesis driven.

### Excellent miRNA multiplexing potential

The data presented in this study were generated using older generation Illumina reagents and flowcells. Using up to date consumables and basecalling algorithms, over 30 million reads per flowcell can be easily be reached. Assuming that approximately 5 million sequencing reads are needed to profile the microRNA transcriptome at sufficient depth, up to six libraries may be pooled and analysed in a single flowcell lane without loss of resolution. The number of sequencing reads is expected to increase in the the near future, making multiplexing of libraries an attractive approach for cost effective smallRNA sequencing. The barcoding system used for the preparation of the libraries in this study were identical to those described in the SREK protocol manual, but an alternative barcoding schemes can easily be implemented. Here we used only the perfect matching barcode reads to sort the sequencing reads to according to their sample identity, leaving a subset of reads un-assigned (data not shown). A more sophisticated method for multiplex barcode sorting, like the Levenshtein distance [33], that takes into account the potential single-base errors that may be introduced during PCR pre-amplification or primer synthesis can be applied in order to decrease the number of un-assigned reads.

## Conclusions

In this paper we present an adaptation to the SREK protocol that reliably generates miRNA sequencing libraries that are compatible with the Illumina Genome Analyzer. Furthermore, we describe a new analysis tool for analysis of next generation miRNA transcription profiling called E-miR. It allows for automated and fast processing of expression data and reports the most commonly used results to the user in a comprehensive expression table and data visualization track files for the UCSC genome browser. Although successfully tested on chicken data, the pipeline can also be used to analyze miRNA data from any species. The main and accessory perl scripts are available via: http://www.lgtc.nl/EmiR.

## Methods

### Tissue harvest and RNA isolation

Fertilized chicken eggs were obtained from a local hatchery (Drost BV, Nieuw Loosdrecht, The Netherlands), incubated at 39°C in a moist atmosphere, and automatically turned every hour. After the appropriate incubation time, embryos were isolated in Earl's balanced salt solution (EBBS, Life Technologies) and staged according to Hamburger and Hamilton [25]. The complete heart tube (HT) was dissected from stage HH16 embryos (EM). Tissue samples were stored at -80°C prior to use. Total RNA and miRNA enriched RNA fractions were isolated with the mirVana*™*miRNA Isolation Kit (Ambion) according to the manufacturers protocol. RNA integrity was confirmed with Agilent 2100 Bioanalyzer pico-RNA and small-RNA chips.

### miRNA sequencing library generation

Sequencing libraries were generated using a modification on the SOLiD Small RNA Expression Kit (SREK) to make it compatible with Illumina Genome Analyzer technology. MirVana-enriched miRNA fractions from HT or EM and EM totalRNA were hybridized and ligated to the A adapter mix to prepare 5' to 3' sequencing libraries. Reverse transcription and RNaseH treatment were as described in the SREK protocol. Library pre-amplification PCR was performed with Phusion Hot Start High-Fidelity DNA Polymerase (Finnzymes) with a set of alternative primers (Figure 1A and Additional file 4 Figure S3) (Integrated DNA Technologies) that contain sequence features compatible with both the adapters that were introduced during the ligation step as well as the oligonucleotides deposited on the Illumina Genome Analyzer flow cell. DNA was denatured for 30" at 98°C followed by 18 cycles with 30" at 98°C, 30" at 65°C, 30" at 72°C and final extension for 5' at 72°C. During amplification, the RNaseH treated cDNA input did not exceed 1% of total PCR volumes. Final primer concentration was 100 nM. All libraries were amplified using a different 6-nucleotide multiplex sequence tag in the 5'-primer. Library fragments were separated on a native 6% gradient pre-cast PAGE gel (Novex, Invitrogen). The 140-155 bp size fractions containing the miRNA inserts were excised, DNA was eluted from the gel, precipitated and dissolved in 15 *μ*L nuclease free water. Library yield was quantified on a Agilent Bioanalyzer DNA1000 chip. Single read cluster generation and single read sequencing were performed according to the standard Illumina v2 and v3 kits, respectively. An alternative set of sequencing primers was used (Figure 1A) during the sequence-by-synthesis steps to determine the RNA insert sequence and multiplex tag. Multiplexed and single read samples were 35 and 32 nt long, respectively. The smallRNA insert and multiplex tags were sequenced using standard Illumina primer annealing protocols. The complete protocol with description of the modifications to the SREK method are available in Additional file 8 Methods S1. These data have been deposited as fastq and wig files in the GEO database as series GSE20757.

### smallRNA sequence data analysis pipeline E-miR

A perl based data analysis pipeline, called E-miR, was built to process the microRNA sequencing data in several automated steps (Figure 2). Multiple data files can be processed in one single run. Multiple input data formats are supported including FASTQ and SCARF as well as a simple tab delimited sequence and counts format. First, the the non-coding transcript annotation files are processed. These annotation files, i.e., non-coding RNA and Dicer processed miRNAs can be retrieved from via the Ensembl perl API with a perl script that is available with the EmiR pipeline. Custom regions of interest may be added to the annotation after these files are downloaded. In EmiR, miRNA transcripts are named according to the 5-prime (5p) or 3-prime (3p) arm of the hairpin they originated from. The relative positions of the 5p and 3p products to the miRNA precursors were and used to recalculated their genome positions. For each miRNA, the two Dicer processed transcripts are processed separately by the pipeline. In cases where relative positions on only one of the 5p or 3p products was available, the complementary positions of the un-annotated transcript were inferred, taking into account the 2 nt overhang of the miRNA duplex. In the second step of the pipeline, the 3' adapter that is introduced during the sample preparation is removed from each sequence by regular expression matching, optionally allowing for one mismatch. Sequences shorter than 15 nt after truncation are excluded from further analysis. The Eland aligner cannot handle sequences longer than 32 nt, therefore, sequences longer that 32 nt are truncated at 32 nt. Next the sum of sequence counts of identical reads after truncation are calculated and separate files for all sequence lengths are prepared for Eland alignment (step 4). All unique sequences from the input samples are aligned to the genome of interest using the Eland alignment tool (step 5). Only sequences uniquely aligned to the genome with a maximum of one mismatch are accepted for further processing. Sequence reads were annotated in step 6 to known non-coding RNA transcript like miRNA, snRNA, snoRNA, tRNA and rRNAs based on overlap of their genomic positions from step 1. In order to handle variations in miRNA transcript lengths, a sequence read is annotated to a transcript locus from the annotation files when there is a minimal 50% overlap on genomics positions. In this step, sequence sets are generated for all transcript regions present in the processed annotation files. During step 7 of the pipeline, all data is compiled into an RNA expression table for all data input files combined. The table contains data for all expressed RNA transcripts as defined in the annotation files. For miRNAs the pipeline distinguishes between the 5p and 3p miRNA precursor sequence products. Previously un-annotated miRNA transcripts generated from known precursors are included as well. MiRNA identifiers are composed of both transcript annotation and genomic location, e.g., miRNA|ENSGALT00000028942 |5p~|gga-mir-29a|sense indicates a miRNA with Ensembl transcript ID ENSGALT00000028942, the 5 prime section of the precursor hairpin of miRNA 29a and the match is in the sense orientation. The '~' in the identifier indicates the positions of this transcript was inferred from the hairpin structure. For each of the expressed RNA transcripts the number of unique reads, representing the number of unique isomirs detected for miRNAs, the total sum of reads, the sum of reads without mismatches and the most abundant transcript are listed. In addition, the sense and antisense orientation of the sequence reads relative to the annotated transcript is reported in the EmiR output in the expression table. To correct for the differences in read counts between libraries, the sum of reads per transcript are scaled to tpm based on the sum of aligned sequence tags. A square root transformation was applied for variance stabilization. For data visualization in the UCSC genome browser files in the Bed (step 9) and Wig (step 10) format are generated. E-miR was designed to run on Linux systems exclusively. On a Ubuntu 9.04 (Jaunty) OS running on an Intel Quad core 2.4 MHz with 4 Gb of memory, analysis of all five libraries described in this manuscript was completed within 2 h and 26 min, 1 hour and 43 min of which were used for the Eland genome alignment step alone. Separating samples based on their multiplex tags is not performed by the E-miR pipeline. Multiplexed sequence reads were separated based on their multiplex sequence reads with a custom command line.

### Statistical analysis of differential miRNA expression

MicroRNA-expression levels were tested for significant differential expression between the HTs and EMs libraries using the Limma [34] package in R. The Benjamini-Hochberg method (BH-FDR) [35] was used to control the false discovery rate. Statistical significance was tested using the square root transformed tpm expression values of the 5p and 3p miRNA transcripts. Differences in expression with BH-FDR p-values ≤ 0.05 and a minimal 1.5 fold change were considered to be significant.

### Isomir analysis

The Globaltest (version 5.1.5) [24] was used to investigate the contribution of each specific isomir to the differential expression of each miRNA. The lists of isomirs per miRNA transcript, i.e., the covariates to be tested, was compiled by the E-miR pipeline (optional feature). An expression table was generated containing square root transformed tpm expression levels for each unique individual isomir transcript. From this table, isomir transcripts containing the 'N' nucleotide were excluded from further analysis. The minimal sum of isomirs for either EM or HT was set at 10 tpm miRNAs with BH-FDR p-values ≤ 0.05 were considered to be significantly differentially expressed between EMs and HTs groups. To avoid mis interpretation due to stochastic sampling effects of less abundant miRNAs, only miRNAs for which the most abundant isomir was above 50 tpm were taken into consideration for further analyses. The differentially expressed isomirs for each miRNA were identified via the 'subsets' and 'leafNodes' functions in the Globaltest package with Holm multiple testing correction p-values at 0.1.

### Quantitative Real-Time PCR

Sequencing derived expression profiles were compared to qPCR measurements on small RNA fractions from independent biological samples. Complementary DNA strands were generated from 300 ng mirVana enriched miRNA fractions in a 20 *μ*L reaction volume using the Invitrogen NCode miRNA First-Strand cDNA Synthesis kit. An equivalent of 0.5 ng RNA was used in the PCR amplification with 100 nM miRNA specific primers (Eurogentec; Additional file 9 Table S4) and 4 *μ*L iQ SYBR Green Supermix (bioRad) in an 8 *μ*L reaction using standard cycle parameters on an LightCycler480 (Roche), with annealing temperature set to 57°C. MicroRNA primers were designed at the 5' regions of the transcripts as to avoid potential mis priming due to the sequence variation observed at the 3' ends of microRNA transcripts. PCR amplification efficiencies for each miRNA transcript were determined directly from the amplifications curves using the LinRegPCR tool [36] and used to calculate relative expression between heart tube and embryo.

## Authors' contributions

HB: microRNA sequencing library preparation, qPCR, data analysis with Limma and Globaltest, designed E-miR analysis pipeline, data interpretation and drafted the manuscript. YA: Illumina GAII sequencer operation, primer design. GJvO: Editing of the manuscript. JTdD: Participated in the design of the study and editing of the manuscript PAH: Participated in its design and coordination, interpretation of the data and helped to draft the manuscript. All authors read and approved the final manuscript

## Supplementary Material

Additional file 1 **Figure S1. Realtime PCR validation of the miSeq libraries**. Realtime PCR validation of the miSeq libraries prepared from heart tube and whole embryo RNA using the modified SREK protocol. Transcript specific forward primers for miR20b-5p, mir-206-3p and miR133 were based on full length miRbase [2] sequences and used in combination with a universal reverse primer that was designed to anneal to the 3' of the miSeq library as indicated in Figure 1A. miRNA expression was expressed relative to the total library content by using a forward primer at the 5' of the miSeq library. Click here for file

Additional file 2 **Table S1. EmiR expression table**. smallRNA expression table as generated by the E-miR pipeline listing all expressed non-coding RNA transcripts from the annotation files that were detected in the sample data. Column description: The first column hold the transcript identifier, e.g., miRNA |ENSGALT00000028942|5p~|gga-mir-29ajsense, indicates the transcript is a miRNA with EnsemblTranscript ENSGALT00000028942 | the 5 prime part of the precursor j transcript name = gga-mir-29a and the match is the sense orientation. In cases where the miRbase has only one of the Dicer cleaved miRNA transcripts, the complementary transcripts were inferred from the hairpin structure. These unlisted transcripts are indicated by a '5p~' and '3p~' in the identifier. Expression for other non-coding RNA transcripts, like snoRNA and tRNA, are also included in the table. All of these have the '|n|' in the identifier instead of the |3/5p|. The second column holds the genome location. For each of the input files, seven columns of data are included, containing the following data: - unique: unique number of reads annotated to this miRNA transcript. - counts: sum of the number of times this miRNA transcript was found. - U0-counts: same as 'counts' but then only the sum of perfect matches only. - highest_count: expression of the most abundant isomer. - highest_seq: Identifier of the most abundant isomir: compiled from chr | begin | end | strand | mismatches in alignment | isomir sequence. - tpm scaled the 'counts' value normalized/scaled to sequences per million. - sqrt square root of the scaled value. This stabilizes variance. Click here for file

Additional file 3 **Figure S2. Embryo small vs total RNA scatter plot**. Scatter plot indicating average miRNA expression in sqrt(tpm) for whole embryo derived libraries generated with small RNA enriched fractions and totalRNA. Open and closed black circles represent non-significant and significantly differentially expressed miRbase miRNA transcripts respectively. The top left insert depicts an enlarged section of the 0-20 sqrt(tpm) area. The table lists the FDR corrected p-value and expression levels in sqrt(tpm) for all six differentially expressed miRNAs. Click here for file

Additional file 4 **Figure S3. Short amplification primers**. Alternative set of short primers used during miRNA sequencing library preparation to make the SREK protocol compatible with the Illumina Genome Analyzer. * Indicates a phosphorothioate bond. Click here for file

Additional file 5 **Table S2. Limma results**. Significantly differentially expressed miRNAs between the Heart and Embryo from the Limma analysis. Columns 1 and 2 are in the same format as those in Additional file 2, Table S1, followed by the average expression levels, in sqrt(tpm), for the EMs and HTs library and BH-FDR p-value for the difference in expression. Click here for file

Additional file 6 **Figure S4. qPCR confirmation of sequencing data**. MicroRNA expression levels for 5p or 3p transcripts in HH16 whole chicken embryo and heart tube. 5 s rRNA was used as an internal control to normalize gene expression. Left and right column represent miSeq and qPCR derived expression levels, respectively. The bars represent mean expression levels ± sd. indicates a significant difference in gene expression relative to whole embryo. Click here for file

Additional file 7 **Table S3. Globaltest results**. Significantly differentially expressed miRNAs between the Heart and Embryo from the Globaltest analysis. Columns 1 and 2 are in the same format as those in Additional file 2, Table S1, followed by the BH-FDR p-value for the difference in expression and the number of isomirs the test was based upon. the last two columns indicate if the miRNA was significant in the Limma analysis and if the most abundant isomir had expression above 50 tpm to be included for further analysis. Click here for file

Additional file 8 **Methods S1. Modified smallRNA library preparation protocol**. step-by-step protocol to generate microRNA sequencing libraries using the SREK kit that are compatible with the Illumina Genome Analyzer. Click here for file

Additional file 9 **Table S4. qPCR primers**. miRNA specific forward primer sequences used for qPCR confirmation of sequencing data. Click here for file
