# Supplementary figures and images for: New methods for next generation sequencing based microRNA expression profiling
Source: BMC Genomics. 2010 Dec 20;11:716. doi: 10.1186/1471-2164-11-716 (PMC3022920; doi:10.1186/1471-2164-11-716)

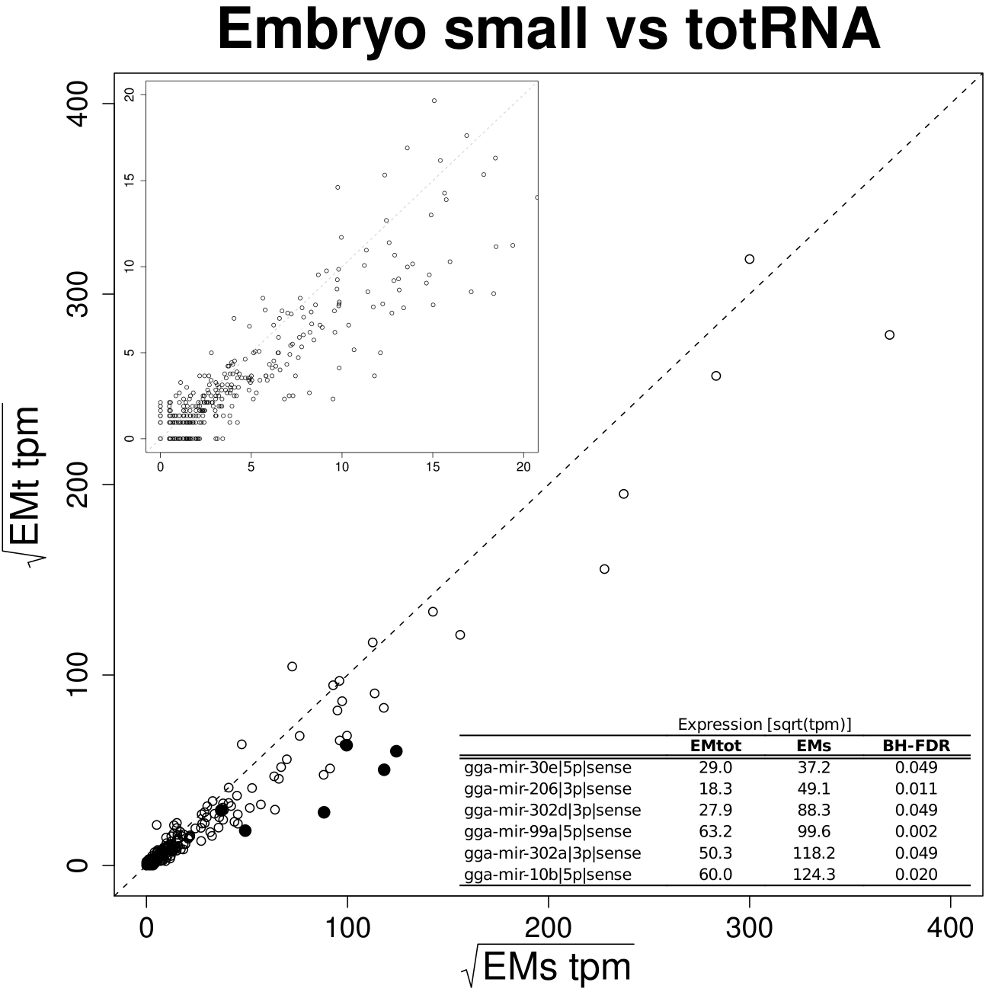

Supplement: Additional file 3 — Figure S2. Embryo small vs total RNA scatter plot. Scatter plot indicating average miRNA expression in sqrt(tpm) for whole embryo derived libraries generated with small RNA enriched fractions and totalRNA. Open and closed black circles represent non-significant and significantly differentially expressed miRbase miRNA transcripts respectively. The top left insert depicts an enlarged section of the 0-20 sqrt(tpm) area. The table lists the FDR corrected p-value and expression levels in sqrt(tpm) for all six differentially expressed miRNAs. [file 1471-2164-11-716-S3.TIFF]

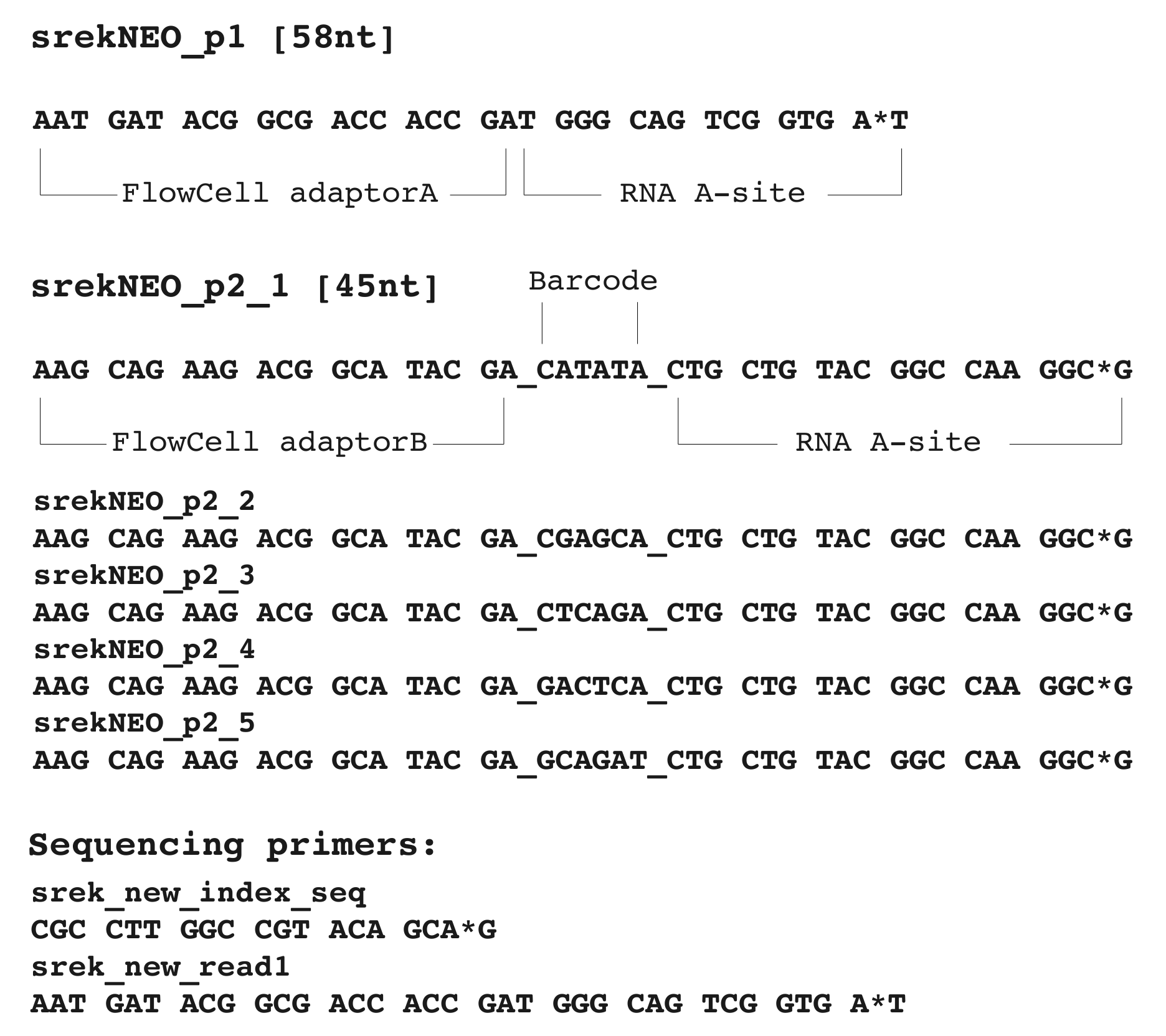

Supplement: Additional file 4 — Figure S3. Short amplification primers. Alternative set of short primers used during miRNA sequencing library preparation to make the SREK protocol compatible with the Illumina Genome Analyzer. * Indicates a phosphorothioate bond. [file 1471-2164-11-716-S4.TIFF]
