# Supplementary material for: New methods for next generation sequencing based microRNA expression profiling
Source: BMC Genomics. 2010 Dec 20;11:716. doi: 10.1186/1471-2164-11-716 (PMC3022920; doi:10.1186/1471-2164-11-716)
Supplement: Additional file 8 — Methods S1. Modified smallRNA library preparation protocol. step-by-step protocol to generate microRNA sequencing libraries using the SREK kit that are compatible with the Illumina Genome Analyzer. [file 1471-2164-11-716-S8.PDF]

Human and Clinical Genetics (LUMC) - LabJ_Protocols©

microRNA sequencing library preparation protocol

Henk Buermans: June 2010

Purpose

This protocol is for preparing microRNA sequencing libraries that are to be sequenced with the Illumina/Solexa Genome Analyzer.
NOTE#1: This protocol described a set of modifications to the Small RNA expression Kit (SREK) from Ambion/Applied biosystems that make this method compatible with the Illumina/Solexa Genome Analyzer.
NOTE#2: Perform cDNA synthesis and assembly of PCR reaction in pre-PCR lab!
NOTE#3: After pre-amplification of the libraries DO NOT continue to work in the pre-PCR room!
NOTE#4: ALWAYS STORE RNA PREPARATOINS AT -80° C
NOTE#5: DURING THIS PROTOCOL, KEEP YOUR RNA PREPARATIONS ON ICE, UNLESS INDICATED OTHERWISE!

Protocol

RNA input:

Sequencing technology is still very expensive. Be sure your input RNA is not degraded! Check it on an agilent nano, pico or smallRNA chip.

It is possible to use total RNA samples. However, a majority of the adapters will then ligate to non-miRNA transcripts like ribosomal RNA. This may impair the detection of low expressed miRNA transcripts in the library. Using *mir*Vana enriched RNA or flashPAGE enriched miRNA fractions is recommended. Sequencing libraries have successful been generated using 500 ng totalRNA and 120 ng *mir*Vana enriched RNA. The protocol allows a maximum of 3µL RNA solution! If needed precipitate the RNA:

- 2 µL (1µg/µL) glycogen carrier or 1 µL Pellet Paint
- 1/10x vol 3 M sodium acetate
- 4 volumes of 100% ethanol

Mix thoroughly and incubate at –20° C for ~16 hr (or overnight). Centrifuge at top speed for 30 min at 4° C. Wash the pellet by adding 500 µL 80% cold EtOH. Centrifuge at top speed for 10 min at 4° C. Make sure the pellet is completely dry before you re suspend the RNA in DEPC-Treated water.

Hybridization and ligation of adapters

In the pre-PCR room prepare the hybridization mix by combining the following components:

- 2 µL Adapter mix A
- 3 µL hybridization solution
- RNA sample up to 3 µL.

Total volume should be 8 µL. Adapter mix A is used to sequence from 5' to 3'. If needed prepare another mixture with adapter mix B to sequence from 3' to 5'.

In a thermal cycler (heated lid) incubate the samples for 10 min at 65° C followed by at least 5 min at 16° C.

Place the samples on ice and ligate the adapters to the RNA transcript by adding the following components:

- 10 µL 2x Ligation buffer. Very Viscous solution. Pipet slowly!
- 2 µL Ligation enzyme mix.

Total volume should be 20 µL per tube. Mix well but carefully and incubate at 16° C for 16 hours (overnight). Set the temperature of the heated lid to match the reaction or turn it off completely.

Reverse transcription and RNase H digestion

Take the samples from the thermal cycler, spin down and add the reagents for the reverse transcription step.

- 13 µL DEPC-treated water
- 4 µL 10x RT buffer
- 2 µL 2.5 mM dNTP mix
- 1 µL ArrayScript reverse transcriptase

Do prepare master mixes for multiple samples here! Total volume should be 40 µL per tube. Mix gently and spin down the tube. Incubate for 30 min at 42° C.

- Add 1 µL RNase H to 10 µL sample, mix well and incubate for 30 min at 37° C.

Only a small amount of the cDNA sample will be used in the PCR pre-amplification. Make aliquots of the remaining RNase H treated cDNA and store at -20 or -80° C.

Library pre-amplification

During the PCR pre-amplification alternative primers are used that introduce sequences to the library that make it compatible with the Illumina/Solexa Genome Analyzer. These primers have sequences that can anneal to the ligated adapter sequences and sequences that can hybridize to the oligonucleotides that are deposited on the flowcell.

The reverse PCR primer also contains a multiplex sequence tag. At the moment we have five different reverse primers available. If you do not intend to use multiplexing, use the same reverse primer for all your samples. Alternatively, use a different reverse primer for each sample. The PCR amplification primers and functional sequences are listed below:

```
srekNEO_p1 [35nt]

AAT GAT ACG GCG ACC ACC GAT GGG CAG TCG GTG A*T
|____FlowCell adaptorA____||____ RNA A-site ____|

srekNEO_p2_1 [45nt]       Barcode
                             |    |
AAG CAG AAG ACG GCA TAC GA_CATATA_CTG CTG TAC GGC CAA GGC*G
|____FlowCell adaptorB____|         |____ RNA A-site ______|

srekNEO_p2_2
AAG CAG AAG ACG GCA TAC GA_CGAGCA_CTG CTG TAC GGC CAA GGC*G
srekNEO_p2_3
AAG CAG AAG ACG GCA TAC GA_CTCAGA_CTG CTG TAC GGC CAA GGC*G
srekNEO_p2_4
AAG CAG AAG ACG GCA TAC GA_GACTCA_CTG CTG TAC GGC CAA GGC*G
srekNEO_p2_5
AAG CAG AAG ACG GCA TAC GA_GCAGAT_CTG CTG TAC GGC CAA GGC*G

Sequencing primers:
srek_new_index_seq
CGC CTT GGC CGT ACA GCA*G
srek_new_read1
AAT GAT ACG GCG ACC ACC GAT GGG CAG TCG GTG A*T
```

['RNA A-site' means RNA adapter site]

Prepare a PCR mastermix with the following components for one reaction (in µL):

	reaction volume	50 µL	100 µL
•	5x PCR buffer	10.0	20.0
•	dNTP (25mM)	1.5	3.0
•	PhusionPolymerase	0.5	1.0
•	H2O	36.7	73.4
	total	48.7	97.4

And add this to another tube containing:

	reaction volume	50 µL	100 µL
•	RNase-H treated cDNA	0.5	1.0
•	srekNEO_p1 & p2NEO_x primer	0.8	1.6

Primer **stock** solutions are 100 pmol/µL. Final primer concentrations should be 100 nM, each. Primer aliquots have been prepared and stored at -20° C (6 µL H2O + 1 µL srekNEO_p1 + 1µL of the p2NEO_x primer) to be used for the specific samples. More cDNA may be added (and use less H2O) but keep in mind that the cDNA sample contains compounds that may inhibit the amplification reaction! Mix well, spin down and setup the following PCR cycle profile:

Denaturation:	30 sec @ 98° C
18 cycles:	30 sec @ 98° C , 30 sec @ 65° C , 30 sec @ 72° C
Final extension:	5 min @ 72° C
hold at 4° C	

Library check

Do this only when you're not sure the RNA input was low! Apply 5% of the amplified library to a native 4-12% gradient PAGE gel and run at 120 V for ~45-60 min or until the lower dye marker has reached the end of the PAGE gel. Be sure to include a size ladder of the appropriate size range! Try to load all well with an equal amount of sample in loading buffer and also load all "empty" slots with 1x loading buffer.

Stain the gel with Sybr-green gold to visualize the fragments. A representative image with the different library fragments formed is shown below:

Multiple bands are visible. When the adapters from the adapter mix A or B self anneal and get PCR amplified, a 80 bp fragment is formed. This fragment does not contain an RNA insert! Adapters can themselves be ligated between adapters and will form the ~90 bp bands. miRNAs range from 15-25 nt in length. The miRNA amplified library size needed is from 95 up to 105 bp, i.e., you'll need to cut out the upper one of the three.

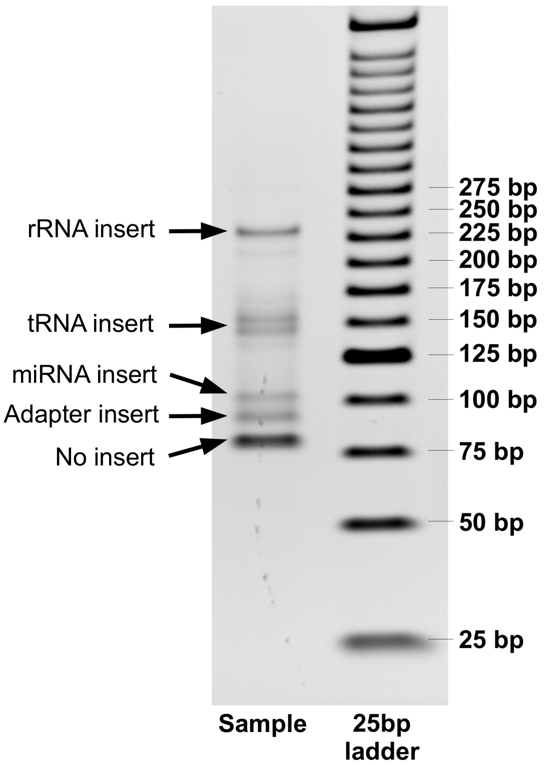

Library size selection and cleanup

Keep the amplified library sample at room temperature. Higher temperatures may melt out the DNA duplexes which will affect PAGE gel migration of the fragments! Use a centrifuge with temperature control.

Phenol:Chloroform extraction

When the library contents have been verified, pool replicate PCR samples and add:

H2O to 500 µL

equal volume (500 µL) of phenol/chloroform/isoamyl alcohol (25:24:1, pH 7.9),

Mix well, keep at room temp for 5 min and spin at 13 krpm for 5 min.

Carefully pipet the aqueous phase (~400 µL) into a new 1.5 mL eppendorf tube and add: equal volume of 5M NH4Ac (400 µL). Total volume should be ~800 µL. Now add 0.7x vol Isopropanol (560 µL) and 1/100x vol Glycogen (8 µL). Keep at RT for 5 min and spin for 20 min @ 13 krpm at RT.

Discard supernatant and wash the pellet 3x with 500 uL 70% EtOH . If the pellet was initially not visible, it will become more white during these washing steps. Be sure to properly dry the pellet before dissolving it in 15 µL 1x Gel loading buffer.

PAGE gel size Selection

Apply the sample in on a 4-12% or 4-20% gradient PAGE gel and load the libraries with at least one lane spacing to avoid cross contaminations. Load a 25bp ladder at convenient intervals to accurately estimate the proper sizes to excise. The size range to excise is 95-105 bp. The Invitogen 10bp bands are close together, may help in the size selection, but mostly look like a smear when observed on the Darkreader. Try to load all well with an equal amount of sample in buffer and also load all "empty" slots with 1x loading buffer. Run the gel at 120 V for ~45 min or until the lower dye marker has reached the end of the PAGE gel. When using pellet paint in the precipitation step here, it does not migrate into the PAGE gel. Stain the gel with Sybr-green gold.

During electrophoresis prepare punctured 0.5 mL tubes and place these in *2.0 mL round bottom tubes*. Excise the correct library fragments and transfer the gel to the 0.5 mL tubes. Use clean blades for cutting of the gel & use each blade ***once*** and discard them immediately after use. Be sure to exclude the lower fragments at ~90 bp as these will also be sequenced but do not contain any useful RNA inserts, thus they lower your sequencing depth! Including higher sizes may also decrease your sequencing depth.

Sred the gel by centrifugation for 3 min at 13 krpm and add 300 µL PAGE elution buffer and incubate at RT for at least one hour with gentle agitation. Overnight is possible as well. Transfer the DNA containing buffer to a clean tube, add 300 µL PAGE elution buffer to the gel fragments and incubate for 1-2 hours at 37° C with gentle agitation. Transfer the gel fragments and all eluted DNA to an Costar® Spin-X® centrifuge tube.

Cut a pipet tip to enlarge the opening may be helpful. Spin for 5 min at top speed to separate the PAGE elution buffer from the gel remains.

Precipitate the libraries by adding to each sample:

1 µL pellet paint

0.7x vol Isopropanol

Mix, leave at RT for 5 min and spin at 13krpm for 20 min. Wash the pellet shortly in 500 µL 70% EtOH. The pellet will become more visible. Carefully dry the pellet and dissolve it in 15-20 µL nuclease free water.

Library Quantification

Apply 1.0 µL per library on a DNA1000 or DNA high sensitivity Agilent chip for DNA quantification and size verification. Do not heat the samples!

Alternatively, qPCR primers that were designed to amplify the whole library, including the small RNA insert, are available to quantify these libraries with the Roche LightCycler 480. Standard SYBR-green-I PCR protocols can be used. Be sure to dilute the libraries 1/1,000 to 1/10,000 as the titers may be very high, resulting in Ct values below 5 cycles, which may not be accurate. Relative library qPCR quantities show high correlations to the DNA1000 chip results. This allows for quantification of new libraries by comparing the relative quantities of new sequencing libraries with high quality libraries that have been proven to yield good sequencing data.

Also if you know that a certain set of microRNAs are differentially expressed between the samples, this can be used to validate the newly generated libraries by using the Library Reverse primer in combination with a specific miRNA forward primer. This mi-primers is the complement of the reverse-transcribed miRNA sequence from the cDNA synthesis reaction, e.g. for gga-miR-499:

miRNA sequence: UUAAGACUUGUAGUGAUGUUUAG
Forward primer: TTAAGACTTGTAGTGATGTTTAG
[substituting U with T should suffice]

This additional QC check is strongly advised to ensure the sequencing library is intact and in agreement with expected miRNA expression profiles. microRNA specific amplicon sizes will be around 98 bp

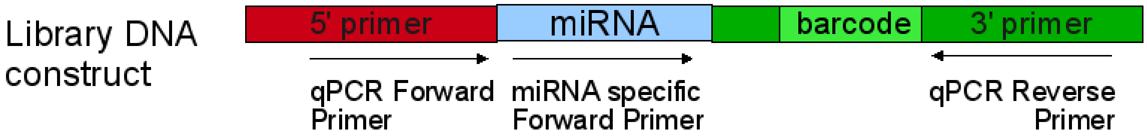

qPCR Forward primer AAT GAT ACG GCG ACC ACC

qPCR Reverse primer AAG CAG AAG ACG GCA TAC

<u>Final library preparation</u>

Prepare a 10 nmol/L sample from the pre-amplified libraries in a 0.1% tween in water solution. If you intend to do multiplex sequencing, ***pool equi-molar*** amounts of each separate library to a total 10 nmol/L concentration in a 0.1% tween in water solution.

The samples are now ready for sequencing.

Reagents that are needed but are not included in the SREK kit:

PAGE Elution buffer. For 10 mL:

  5 mL TE buffer (10 mM Tris-HCl, pH8.0, 1mM EDTA)

  5 mL 5 M NH4Ac

70% & 100% EtOH

100% Isopropanol

Phenol/chloroform/isoamyl alcohol (25:24:1, pH 7.9)

Glycogen (1 µg/µL) or Pellet Paint

Novex precast 4-12/20% PAGE gels

1x TBE buffer

Costar® Spin-X® centrifuge tubes

Clean Razor blades
